# Cecal microbiota transportation, social stress, and injurious behaviors in chickens – A review

**DOI:** 10.1016/j.psj.2026.107305

**Published:** 2026-06-18

**Authors:** Heng-wei Cheng

**Affiliations:** Livestock Behavior Research Unit, USDA-ARS, West Lafayette, IN 47907, USA

**Keywords:** Social stress, Injurious behavior, Intestinal microbiota, Microbiota-gut-brain axis, Chicken

## Abstract

Aggression and injurious behaviors, including aggressive pecking, feather pecking, vent pecking, and cannibalistic pecking, pose significant health, welfare, and production challenges for laying hens and the global poultry industry. Despite the technological advancements in poultry farming practices, injurious behaviors occur in all the current housing systems. Evidence from human and laboratory mammalian studies suggests that early-life stress may impair gut microbial development and affect gut-brain axis signaling through changes in gut permeability and inflammatory pathways, resulting in neuroinflammation. As a consequence, brain damage causes mental and emotional disorders with abnormal behaviors. Therefore, intervening for maladaptive social behaviors has become a critical topic in various animals including chickens. Despite continuously evolving the outcomes of the microbiota-gut-brain axis interplay, whether similar mechanisms contribute to maladaptive or injurious behaviors in chickens remains an important area for further investigation. The reviewed issues provide an exhaustive scientific analysis of the activation of cecal microbiota transplantation in poultry and describe a potential that injurious behaviors in chickens can be reduced by modification of their gut microbiota profiles and brain serotonergic activity. The outcomes provide a novel insight into developing an intervention strategy for preventing or inhibiting injurious behaviors in chickens. To fully test the strategy, a comprehensive study is required to further explore the signaling mechanisms of cecal microbiota transplantation in injurious behaviors and its scope of application, especially in laying hens.

## Introduction

The conventional cage systems, also referred to as battery cage systems, are currently among the most commonly used housing systems in global egg production, including the United States. Approximately 60.5% of 311million commercial laying hens in the United States live their entire lives in the conventional cage systems (UEP, 2026). Despite the intensified system successfully prioritizing hygiene, disease control, and economic output, its high stocking density with a barren environment restricts natural behaviors in chickens, such as foraging, dustbathing, preening, walking, running, and perching, causing maladaptive behaviors, alongside physiological manifestation of chronic social stress (Alig et al., 2025; Hofmann and Stefanski, 2025). Injurious behaviors in chickens, such as aggressive pecking, feather pecking, vent pecking, and cannibalistic pecking, are seriously unsolved health, welfare, and production issues. Although currently developed operational management, such as optimizing nutrition and lighting, offering environmental enrichment, and providing adequate space, beak trimming (i.e., removal of 1/3 to 1/2 of the beak) is the most effective and reliable practice for reducing the harmful behaviors in laying hens (UEP, 2025). However, beak trimming is a controversial subject since the avian beak is a multipurpose functional organ playing a vital role in feeding, drinking, preening, and mating as well as defensing predators and parasites (Iqbal and Moss, 2021). Recent studies have indicated that compared to hot blaze beak trimming, infrared beak treatment is a less painful procedure with multiple advanced benefits, such as less impacting feed intake, drinking, growth, and engagement in some natural behaviors (Dennis et al., 2009; Struthers et al., 2019). However, regardless of the methods used, beak trimming causes tissue damage and nerve injury, subsequently leads to pain (acute, chronic, or both) and functional limitation negatively impacting chicken welfare (Kuenzel, 2007; Gentle, 2022). In contrast, omitting beak trimming results in increased incidence of harmful behaviors and potential negative consequences for skin wellness, production, and survivability in chickens (Riber and Hinrichsen, 2017; Austry, 2022). Recent studies also revealed that injurious behaviors still occur in beak trimmed flocks irrespective of housing systems, including cage (conventional and enriched cages), cage-free (aviary systems with single- or multi-tiers), and organic operations (Schwarzer et al., 2021, 2022a,b). With passed legislation at several states and pledged by multiple retailers and food service providers to ban confinement hens, hen production in the United Sates is transiting from conventional cage systems (a cage with a capacity of 5-9 hens, 67-82 sq. in. of usable space per hen) to cage-free systems (a barn with a capacity of 100,000 birds or more, 0.0929-0.1394 sq. m. of usable space per hen) (Caputo et al., 2023; Ellis, 2025). Despite cage-free systems providing some benefits to hens, such as nesting, scratching, dustbathing, and perching behavior, the larger group size often leads to higher risk of harmful behavior, resulting from increased social pressure (i.e., social influence or dynamics with lacking cohesion). Chickens are social animals, similar to humans, thriving to live in a group to learn from each other; however, individual birds may experience stress due to interactions with unfamiliar flock members, causing potential interaction-based threats (Nicol and Pope, 1999; Nicol, 2006; Nicol et al., 2011). A hen can only recognize approximately 100 different individuals within a flock up to 30 days, and unfamiliar birds perceive threats during the establishment of dominance interactions (Bradshaw, 1991, 1992; Vallortigara et al., 2010; Rugani et al., 2015).

Social stress in animals has been catalyzed as a state, i.e., psychological and/or physiological dysregulation, raised from one’s relationships with others within a group or flock environment (Karpinski et al., 2018; Barnett, 2023). Psychological dysregulation refers to difficulty managing one’s emotional response to a given situation, triggering fear, depression, and/or anxiety (Wu et al., 2024), whereas physiological dysregulation often occurs as the autonomic nervous system fails to maintain the internal homeostasis in responding to stimulation, leading to excessive sweating, rapid heartbeat, and/or fast breathing (Lu et al., 2021). Generally, stressful environment within a society is a critical factor causing biological allostatic overload, alongside intergroup conflict and violent exchange (De Dreu et al., 2022; Meidenbauer et al., 2025), especially during the late prenatal and early postnatal biobehavioral developments in humans (Nakama et al., 2023; Nolvi et al., 2023; Marceau et al., 2024). Those life developing stages characterized by extensive neurogenesis and functional plasticity (programming and reprogramming of neurobehavioral circuits) are extremely sensitive to internal and external stimulations (Malave et al., 2022; Nicolaides et al., 2024: Donati et al., 2026). In chickens, similar to in humans, exposing themselves to stressful environments during early life has become one of the major reasons for causing injurious behaviors (Elfwing et al., 2015; Hofmann and Stefabski, 2025). Emerging studies have indicated that stressful risk and potential suffering in socioenvironmental context, i.e., social safety (friendly social bonds) and social threat (intergroup competition) theories are presented between humans and other social animals (Ferdowsian and Merskin, 2012; Fields, 2019; Barclay and Benard, 2020), and antisocial personality traits transcend multiple species (Hopwood and Bleidoen, 2021; Black 2025). In chickens, injurious behaviors exhibit a broader phenotype, i.e., a subset of individuals showing great antisocial personality, with a gene-environmental interaction (Johnsson et al., 2018; van der Eijk et al., 2018; Tiemann et al., 2022). Studies also revealed that intestinal microbiota composition and diversity are linked to personality traits (Johnson, 2020; Rangraze, 2025), playing a critical role in maintaining a host’s health (Talate et al., 2025; Wang et al., 2026), while dysfunction of microbiota (alteration of microbiota diversity, composition, and/or function) is a risk factor heightening stress response with mental disorders across species from humans (Dziedziak et al., 2025: Kamath et al., 2025; Ataei et al., 2026; Sie and Tropini, 2026) to chickens (Fu et al., 2024; Gupta et al., 2025).

Gut microbiota functions as an endocrine organ playing critical roles in early brain programming, especially in the development of the “emotional” brain circuits, including the hypothalamic-pituitary-adrenal (HPA) axis and the serotonergic-dopaminergic system governing reward-, stress-, and fear-related behaviors in responding to internal and external stimulations in humans and rodents (Jameson and Hsiao, 2018; Mbiydzenyuy and Qulu, 2024; Diotaiuti et al., 2025; Ramadan et al., 2025; Barakat et al., 2026). By contrast, stress-induced intestinal dysbiosis releases various neurotoxic compounds, consequently, causes neuroinflammation and leads to pathophysiogenesis in patients with various mental disorders (Madison et al., 2024; Schoultz et al., 2025; Nayak et al., 2026). Especially, adversity during early life blunts intestinal microbial colonization, distribution, and function, influencing the development of brain structure and function through the gut-brain axis, subsequently causing long lasting adverse outcomes including increased frequency and intensity of maladaptive behaviors (Mulder et al., 2024; Borrego-Ruiz and Borrego, 2025; Tarracchini et al., 2026). Similarly, early life adversity in chickens disrupts gut microbial development and its links with the central nervous system, impairing development of brain architecture and function in cognitive flexibility, subsequently, reducing inhibitory control in fearfulness (van der Eijk et al., 2020; de Jong et al., 2022), however, it is affecting mental health and injurious behaviors in chickens has not been well examined. Taken together, studies have revealed that the mental health or social wellness of social animals within a group (or a flock) is associated with individuals’ differences (phenotypes) in their physical characteristics, behavioral traits, and biological properties including the intestinal microbiota diversity, composition, and function across species (Marino, 2017; Fulton et al., 2025; Guan et al., 2025; Orme et al., 2025; Ye et al., 2026). Several bacteria, such as *Lactobacillus* and Bifidobacterium, *exert protective effects within individuals experiencing mental disorders by modulating gut microbiota composition, enhancing beneficial bacteria while suppressing harmful pathogens (*Aizawa et al, 2019*;* Matera, 2024*;* Harsa et al., 2025*;* Kaur, 2025*).* Targeting the correlation between personality and microbiome for reducing mental disorders has become a critical topic in humans (Mikami et al., 2023; Tang et al., 2025; Ataei et al., 2026) and various animals including chickens (Yue et al., 2024; Ren et al., 2026; Zhang et al., 2026).

Currently, using probiotics, synbiotics, and psychobiotics for restoring imbalanced microbiota diversity and composition in early life has become a biotherapeutic strategy in treating patients with neuropsychiatric disorders (Kezer et al., 2025; Smolinska et al., 2025; Sun et al., 2025; Venkataraja et al., 2026). Similarly, in chickens, modification of gut microbiota has become an effective management practice for optimizing poultry production via promoting beneficial bacteria and suppressing pathogens to improve gut health, nutrient absorption, and immune function (Yue et al., 2025; Zhang et al., 2025; Li et al., 2026; Yu, 2026). Several probiotics and synbiotics, such as *Lactobacillus, Bifidobacterium, Bacillus subtilis*, and *Saccharomyces,* have been used as natural alternatives to antibiotics (Yaqoob et al., 2022; Hosseini et al., 2026; Khalid et al., 2026;) to control the risk of *Escherichia* and *Salmonella* infection (Dushayeva, 2025; Adeyemi and Nahashon, 2026; El-Shall et al., 2026), *Campylobacter* colonization (Zefanias et al., 2026), tick-borne disease (Tonk-Rugen et al., 2026), and Coccidiosis (Roslon et al., 2025). Beyond those biological benefits, probiotics, such as *Bacillus subtilis* and *Lactobacillus rhamnosus*, have been shown to influence behavioral patterns, particularly by reducing stress and aggression in laying hens through modification of the serotonergic system (Mindus et al., 2021; Jiang et al., 2022; Idowu et al., 2025; Wu et al., 2026). Despite continuously evolving the outcomes in the microbiota-gut-brain (MGB) investigation, there is still a critical need for understanding the function of the MGB axis in host health and illness, especially in chickens. To date, preventing or reducing injurious behaviors to ensure animal health and welfare is a critical challenge facing the poultry industry.

### Stress and injurious behaviors in laying hens

Commercial laying hens have been selected for high production performance, i.e., egg production and feed efficiency, to meet the increasing demand of animal-derived products for global population established to reach approximately 10 billion by 2050 (Kleyn et al., 2021). Selective breeding of animals has shaped biological, morphological, and behavioral traits that facilitate their adaptation to artificial housing environments (Lee, 2021; Gjoen and Jensen, 2024). However, based on the whole organism theory recognizing the internal ecology of an animal’s structural and functional interrelation as a whole (Wilks, 2023), the selection focused on a single production trait can significantly impact other physiological and behavioral trails. These changes may increase the animal’s competition and aggression (Sturmey, 2022; Philson et al., 2025). The study outcomes focusing on injurious behaviors in chickens support the hypothesis that the productivity of an animal is correlated with its competitive ability within a flock (Dale and Craig, 1959; Nikolov and Kanakov, 2022; Wu et al., 2023; Choi et al., 2026). Aggressive behavior in birds, like in humans and other social animals, has functions during social interactions to protect essential resources, such as food and water, associated with the animal’s successful survival, growth, and reproduction; while simultaneously it has become major health and welfare issues since that social instability increasing interspecific competition and stress susceptibility with damaged cognitive abilities and elevated fighting, fear, anxiety, panic, injury, cannibalism, and mortality (Hocking 2014; Arnould et al., 2024; Lowney and Thomson, 2026). Although the current advances in husbandry practices for chickens to combat stress and stress-induced abnormal behaviors, such as developed enriched cage and cage-free aviary systems, poultry egg production is still impacted by environmental stressors associated with the current housing systems (Campbell et al., 2022; Erek and Matur, 2024; Ncho et al., 2025) as that chickens have a social hierarchy to establish dominance rank, also known as pecking order, within a flock (Tibbetts et al., 2022; Grethen et al., 2023). In addition, based on the concept of learning and/or rewarding behaviors, similar to the mechanisms of operant conditioning in humans (Zimlich, 2026), initiated injurious behaviors by a few chickens can be spread throughout entire flock after peckers eating flesh (cannibalistic pecking flesh from a living fowl) (McGrath et al., 2016; Bardach and Murayama, 2025). Chickens without adapting to their rearing environments enter a “pathological state” with atypical responses (hypo- or hyper-reactivity) through activating the stress regulating pathways (i.e., both the HPA axis and sympathetic-pituitary-adrenal axis) and/or the enforced reward and control circuits, i.e., the pallium-ventral tegmental area-medial striatum subregions-posterior pallial amygdala complex circuit to dopamine-like-intrinsic pathways (Acerbo et al., 2002; Rose, 2022; Tian et al., 2022; Gunturkun et al., 2024). The pallium in birds is the telencephalic region functionally analogous to the mammalian cortex (Jarvis, 20095-HT; Smulders, 2021; Zaremba et al., 2025).

**Fig. 1:**
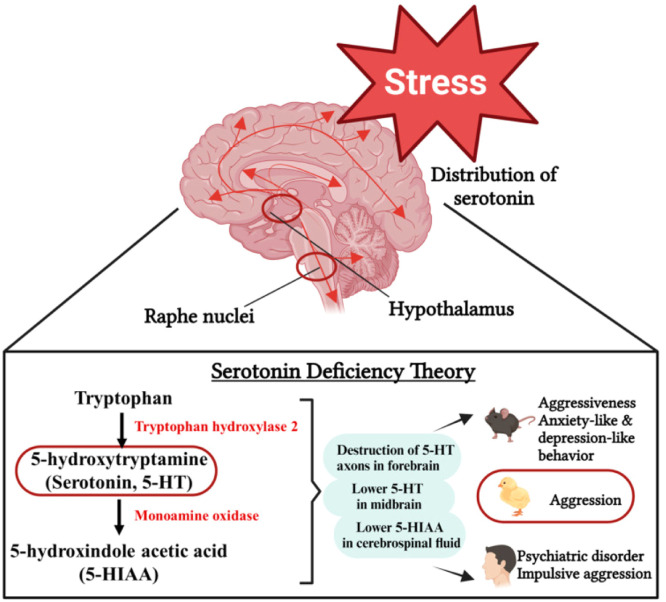
Stress-induced serotonin deficiency theory (Prepared with BioRender.com. Fu and Cheng, 2024).

Generally, chickens endure various stressors raised from their environments daily, such as thermal stress (hot or cold) (Hossain et al., 2026), physical stress (isolation, overcrowding, or nutritional deficiency) (Sugiharto, 2022; Bai et al., 2026), environmental stress (noise, air quality, intensive lighting, or barbaric living conditions) (Kang et al., 2023; Jeon et. Al., 2025 Huang et al., 2026), and biological stress (social unstableness, fear, inflammation, or infection) (Carvalho et al., 2018). The outcomes in chickens, like in other vertebrates (Bolton et al., 2019; Herzberg et al., 2025; Pena, 2026), could be a positive (eustress) or negative (distress) impact on their health based on the type of stressors (pleasure vs. unpleasure) as well as their frequency (single vs. repeated), duration (acute vs. chronic), intensity (mildly vs. severe), and combination, especially during the late prenatal and early postnatal developments with extensive neuroplasticity (Davidson and McEwen, 2012; Drummond and Ancona, 2015; Wada and Coutts, 2021; Hassaneen et al., 2025). Exposed to stress during early life impairs brain programming (altering neurogenesis, synaptic formation and connection, and signal transmission), such as the development of the pallium-hypothalamic-amygdala axis and the serotonergic-dopaminergic circuit (Metwalli et al., 2023; Tamta et al., 2023). Dysregulation of the HPA axis (a major stress responding system also functionally affecting mental health) and the central serotonin (5-HT) deficiency (a low brain 5-HT causing an imbalanced neurotransmission) are strongly associated with a wide range of mental or emotional disorders, including depression, anxiety, and cognitive decline across species, including chickens (Wang et al., 2014; Phi-Van et al., 2018; George et al., 2025; Lei et al., 2025; Lyonna et al., 2025) (Fig. 1). Both systems have become an integrated brain center for aggressive investigation.

Chickens are social animals with cognitively, emotionally and socially complex as mammals (Marino, 2017). Numerous studies have revealed that the neural circuit for aggression and social behavior conserved evolutionarily across the vertebrates (Lischinsky and Lin, 2020). A “core aggression circuit or social behavioral network” including the basal forebrain, midbrain, and mesolimbic system involved in mental and emotional flexibility is present in the brain of mammals and birds (Haller, 2020; Hecker et al., 2025; Li et al., 2025; Santin, 2025). In addition, brain 5-HT actively inhibits excitatory neurotransmission and modulates emotion and mood, nociception, and aggression across multi-species (Yamamoto et al., 2022; Ogelman et al., 2024; Zheng et al., 2024; Chang et al., 2026). In chickens, these nuclei exert similar cognitive function with the capability of plasticity in responding to various environmental stimulus (Drummond and Ancona, 2015; Pozner et al., 2018; Gunturkun et al., 2024), and studies have revealed that birds have similar distributions of 5-HT neurons and 5-HT receptors as those in mammals (Huang and Cheng, 2022; Fujita et al., 2022, 2023). The 5-HT deficiency is the major theory of injurious behaviors in birds (de Haas and van der Eijk, 2018; Tricklebank and Petrinovic, 2019; Bacque-Cazenave et al., 2020). In chickens, increased dietary 5-hydroxytryptophan (5-HPT), an intermediate between tryptophan (TRP) and 5-HT synthesis, reduces fearfulness in a tonic immobility test (Lundgren and Lovlie, 2023). The test is routinely used for assessing fear response in birds (Woodruff, 2025). In addition, prenatal 5-HT fluctuation (as a biological imprinting during a key embryonic developmental stage to activate a specific physiological system to create a lasting pattern in its function) by injection of exogenous 5-HT (20 µg/fertilized egg) to embryos immediately before incubation increases the development of serotoninergic neurons and related neural circuits (Huang et al., 2021), resulting in reduced injurious behaviors in chickens (Huang et al., 2023). Injurious behaviors in chickens can also be reduced via embryonic exposure to TRP (500 µg/embryo at E12), a precursor of 5-HT, via reprogramming the serotonergic system and the MGB axis (Huang and Cheng., 2022). Furthermore, Fortunato et al. (2025) reported maternal TRP supplementation alters the development of the gut-brain axis and social behavior in offspring. Sun et al. (2025) also reported that prenatal high-fat diet disrupting TRP metabolism causes social behavioral deficits in offspring with long-last effects. Recently, it has been discovered that probiotics, such as *Bacillus subtilis,* can prevent aggression in chickens via modifying the central serotonergic system through the MGB axis (Chen et al., 2022; Jiang et al., 2022; Gao et al., 2025; Idowu et al., 2025).

### Intestinal microbiota and the microbiota-gut-brain axis in responding to stress

Increasing studies have reported that microbiota serves as a critical behavioral regulator in personality traits, especially during early-life when the co-developing stage of the brain neurons and gut microbiota is presented (Lynch et al., 2023; Zhu et al., 2025). Generally, the intestinal microbiota functioning as ‘an endocrinological organ’ and ‘a second brain’ impacts host health via multiple biophysiological routes, such as regulating food digestion, nutrient absorption, pathogenic colonization, intestinal epithelial and biochemical barriers, immunity, neurocognitive function, and synthesis of bioactive factors, including short chain fatty acids (SCFAs) and TRP, to maintain biological homeostasis through various ‘gut-organ’ axes (Pires et al., 2024; Ray et al., 2025; Zhao et al., 2025; Gou et al., 2026; Zuo, 2026). Tryptophan, an essential dietary amino acid, is produced within the gastrointestinal tract (GIT) through food intake via the microbiota-tryptophan axis (Gao et al., 2020; Xia et al., 2026) and then enters the brain through the blood-brain-barrier for synthesizing 5-HT via tryptophan hydroxylase-2 within the raphe 5-HT neurons (Zhang et al., 2004; Hoglund et al., 2019). Both TRP and 5-HT have functions in treating mental disorders including anxiety and depression in humans (Kikuchi et al., 2021; Davidson et al., 2022; Adkunle and Balogfun, 2025; Bautista et al., 2025). Acute TRP depletion, such as corn feeding without TRP supplementation, reduces 5-HT neurons in the raphe nuclei (Orozco-Suarez et al., 2003), lowers central 5-HT concentrations (van Donkelaar et al., 2011), and increases aggression (Young, 2013); while feeding TRP diminishes aggressive behaviors by centrally enhanced serotonergic neuronal firing and gut microbiota derived TRP metabolites (Galligan, 2018; Kern et al., 2025) across multiple species, including humans (Zamoscik et al., 2021), pigs (Henry et al., 2022), mice (Walz et al., 2013; Zhang et al., 2026), dogs (DeNapoli et al., 2000), and chickens (Shea et al., 1991, 1996; Poule.net, 2025). In addition, chronic stress disrupts TRP metabolic balance between the tryptophan-serotonin and tryptophan-kynurenine pathways (Li et al., 2025), increasing kynurenine (KYN) has great risk of neurodevelopmental disorders in offspring. Studies has revealed that the offspring of KYN-treated mice produce various diseases including neurological disorders and mental illnesses with behavioral abnormalities (Murakami et al., 2021; Wang et al., 2025). In addition, current studies have further revealed that gut microbiota has significant influence on social behavior and aggression via regulating activation of the HPA axis (Langmajerova et al., 2023; Charalambous et al., 2024; Griffiths et al., 2025). Dysfunction of hypothalamic circuits associates with stress-disrupted serotonergic tonic control, altering HPA function to increase the risk of social behavioral disorders (Bertollo et al., 2025). Animals predisposed to dominating conspecifics often have a low level of 5-HT with high CORT concentrations (Summers and Winberg, 2006). In addition, excess administration of glucocorticoids during fetal development stimulates aggression in rodents (Hayden-Hixson et al., 1991) and chickens (Ahmed et al., 2014).

The differences in gut microbial taxa have been recognized as the major reason for subsets of individuals who are more sensitive to stressful stimulations even under the same or similar living conditions (Iimura et al., 2023; Rangraze et al., 2025); while the dysfunction of gut microbiota (changes in composition and/or synthesis of metabolites) is involved in developing mental disorders in multiple animals including chickens (Fu and Cheng, 2024; Wang et al., 2024; Ataei et al., 2026; Espinosa et al., 2026; Ghahari et al., 2026; Wu et al., 2026). In chickens, current studies have revealed that the levels of injurious behaviors are correlated with the differences in the gut microbiota diversity and/or composition (Meyer et al., 2013; Birkl et al., 2018; Gao et al., 2025). van der Eijk et al. (2019) investigated the differences in gut microbiota composition between laying hens divergently selected on feather pecking. Compared to low feather pecking birds, high feather pecking birds had a higher diversity and evenness of cecal microbiota at adult age (30-wk-old). Especially, high feather pecking birds had a higher abundance of Clostridiales but lower abundances of *Staphylococcus* and *Lactobacillus*. Similarly, Birkl et al. (2018) reported that compared to low feather peckers, high feather peckers had a reduced overall beta microbial diversity with a more abundance of Clostridiae but a lower abundance of Lactobaccillacae*.* Hu et al. (2022) revealed that diversely selected laying hens (Lines 6_3_ and 7_2_ selected for resistance and sensitive to Marek’s disease, exhibiting low and high aggressiveness, respectively) had different cecal microbiota compositions. Line 6_3_ birds had enriched *Faecalibacterium, Oscillibacter, Bautryicicoccus*, and *Bacteriodes* with higher central 5-HT and TRP levels, while line 7_2_ birds had enriched *Clostridials vadin BB60, Alistipes*, and *Mollicutes RF39* with upregulated kynurenine pathway*.* In addition, laying hens (75-wk-age) showing different fear responses during tonic immobility test have different gut microbiota composition with relative immune dynamics (Wang et al, 2024). Birds highly responding to the test have a more enriched gut microbiota with a sensitive immunity. Furthermore, high and low feather pecking chickens also have unique line’s gut microbial metabolite profiles and immunity (Wang et al., 2023; Hofmann et al., 2025). Pekers have relative abundance of *Bacteroides* and *Genniger* in the cecum, positively correlated with plasma L-TRP (the proteogenic, biologically active form of TRP) as well as beta-tyrosine and L-histidine levels, and exhibiting stress-induced immunosuppression with increased plasm corticosterone and norepinephrine levels, compared to neutrals (Wang et al., 2023). These studies indicate that gut microbiota plays a critical role in chicken health, while stress induced gut dysbiosis causes pathophysiological alteration, leading to various disorders including displaying abnormal behaviors, especially during prenatal and postnatal developmental periods with life-long effects. In early life, various probiotics and synbiotics have been used as growth promoters and immune stimulators to replace antibiotics in poultry production (Halder et al., 2024; Naeem et al., 2025; Hassan et al., 2026; Pongmanee et al., 2026). To enhance gut microbial development, in ovo injection of living bacteria (probiotics or synbiotics) and neuro- or immune-stimulators (prebiotics and chemicals) has been developed for improving growth performance and intestinal health as well as stress reactions in chickens (Mangan et al., 2025; Rowland et al., 2025). Therefore, similar in humans, modifying or restoring gut microbiomes has become a novel strategy for preventing or inhibiting injurious behaviors in chickens. As evidence mounts, the use of personalized psychobiotics (next-generation probiotics) (Rosas-Sánchez et al., 2025; Lakshmi et al., 2026) as well as microbiota transplantation (such as fecal microbiota transplantation, FMT) (Zhang et al., 2025; Cao et al., 2026) to restore stress-induced dysbiosis have offered novel therapeutic strategies in treating patients with neuropsychiatric disorders.

Several gut microbiota transplants in chickens, like FMT in humans, have been developed as novel methods for mitigating stress-induced pathogenic bacterial colonization and associated negative effects on production, health, and welfare, via the gut-brain axis (Fu et al., 2024; Liu et al., 2025; Zhao et al., 2025). In humans, FMT (a stool transplant) is an approved treatment for *Clostridioides difficile* infection (Hou et al., 2025; Novelle et al., 2025). The aim of the process is transferring fecal bacteria as well as other microbes from a selected healthy individual (a donor) to a patient with intestinal diseases (a recipient) to replace the pathogenic bacteria with beneficial bacteria, restoring and/or rebalancing gut bacterial diversity and composition. Human FMT studies have established a clear link between the gut microbiome and observed impact on personal phenotypes. Cao et al. (2025a,b) investigated the potential causal relationships between gut microbiota and depressive phenotypes. In the study, fecal samples from women with prenatal depression were transplanted into germ-free mice and revealed decreased gut *Ligilactobacillus* while increased *Akkermansia* in FMT mice with depressive-like behaviors alongside increased plasma LPS and hippocampus neuroinflammation compared to controls (received fecal samples from healthy matched women). In chickens, FMT has also been used to restore gut microbiota balance for reaching an optimum production. Gao et al. (2025) reported that recipients (75-wk-old non-laying hens, laying rate = 0%) received fecal samples from donors (75-wk-old high-yield hens, laying rate > 90%), resulting in increased *Bacteroides* in the cecum similarly to that of donors, with improved egg production. Xu et al. (2025) also reported that FMT significantly altered the gut microbiota and enhanced growth performance in recipients. Two unique fecal samples were used in the study, i.e., the fecal samples from fast-growth chickens had abundant *Lactobacillus* while slow-growth chickens had a higher abundance of Bacteroidetes. Following FMT from fast-growth donors, it significantly increased *Lactobacillus* abundance and glutamine levels promoting myoblast proliferation and differentiation and increasing growth performance in slow-growth recipients. However, FMT outcomes in chickens, as in humans, are affected by multiple factors, including the practical protocols (such as donor variability, amount of fecal dosage, route of delivery, frequency of infusions, and adjuvant treatments), the microbial behave (differently in the recipient’s intestinal tract affected by feed-, geography-, and management-based effects), and pathogen transfer with the procedure (Yadegar et al., 2024; Jankowski et al., 2025). To further investigate the effects of microbiota on chicken production and health, cecal microbiota transplantation (CMT) has been developed with the focus on developing of next generation probiotics, psychobiotics, and or psychobiotic compounds, which may be used without those limitations (Fu and Cheng, 2024; Fu et al., 2024; Khalid et al., 2024).

The avian cecum as a multi-purpose organ has a particularly important role compared with the cecum in other vertebrates (Amir, 2022; Di Marcantonio et al., 2022). Along the GIT, the avian cecum harbors extensive bacterial diversity with a complex community (Polansky et al., 2015), i.e., *Firmicutes, Bacteroidetes*, and *Proteobacteria* at phyla level and *Oscilospira, Bacteroides, Helicobacter*, and *Lactobacillus* at genus level, as the most dominated microbiota, in adult chickens (Ijaz et al., 2018; Cardenas et al., 2021). Functionally, except water and mineral absorption, the cecum is involved in bacterial fermentation and synthesis of various neurotransmitters (such as 5-HT, dopamine, and gamma-aminobutyric acid) and bioactive factors (such as short-chain fatty acids, SCFAs) to regulate mucosal barrier, nutrient digestion and absorption, and immunity (i.e., immune surveillance, clearance of pathogens, and acute phase response) (Jozefiak et al., 2004; Lyte et al., 2022; Dezfouli et al., 2024). In addition, as an out pocket of the intestinal wall at the ileocolic junction, the cecum has two openings into the ileum and the colon. It functionally releases cecal contents into the ileum providing bacteria to maintain gut microbiota diversity and composition (Clench and Mathias, 1995). Like in mammals, chicken cecum is also sensitive to various stressors with reactions appearing to gene-environment interaction (Virolainen et al., 2023). The studies have revealed that chicken lines (breeds) with unique cecal microbiota composition (Yan et al., 2023; Jian et al., 2025) are responding differently to environmental simulations (Bari et al., 2022; Campos et al., 2023) and to experimental challenges with *Salmonella* or *Eimeria necatrix* (Cazals et al., 2022; Wang et al., 2024; Xue et al., 2025). In addition, 1-day-old broiler chicks (recipients) that received cecal contents from hens (donors at 40-wk-old) have bacterial profile similar to the cecal microbiota profile of the donors (Marcolla et al., 2023) with increased beneficial taxa abundance (Khalid et al., 2024). Glendinning et al. (2022) investigated effect of CMT between different broiler breeders during the early-life of chicks. Newly hatched Ross 308 broiler chicks (within < 12 h post-hatch) received cecal samples from adult Roslin broilers (40-wk-old) had higher different cecal microbiota composition and diversity at day 7 post-hatch than control chicks received phosphate-buffered saline (PBS). In a two-week trial, day-old broiler chicks (recipients) received a single passage of the pooled cecal contents from 6-wk-old donor broilers have higher diversity and species richness with increased abundance of the bacterium *Faecalibacterius prausnitzii* during the 1^st^ week of life, effectively against colonization and shedding of *Salmonella Typhimurium,* compared to PBS controls (Pottenger et al., 2023). Similarly, Ramirez et al. (2020) investigating the effect of microbiome transplant-induced cecal community dynamics and phenotypes in broilers reported a differentially abundant taxa of microbiota in recipient (day-old chicks) derived after a single CMT from donors (6-wk-old commercial broiler chickens after a serial transferring of cecal material through multiple generations) at day 14 compared to PBS controls. The recipients also showed protective effects against pathogenic challenge (*Salmonella enterica subsp enterica serovar Typhimurium* and a *Campylobacter jejuni* strain). Microbial functional analysis also shows that day-old chicks (recipients, Lohmann Pink breed) received cecal microbiota from healthy old hens (donors, 47-wk-old Lohmann pink laying hens at a high egg production stage) have different gut microbiota composition and diversity, i.e., increased relative abundances of *Bacteroides, Rikenellaceae_RC9_gut_group*, and *Prevotellaceae_UCG-001* (some SCFA-produced bacteria) while reduced relative abundances of *Alistipes, Lactobacillus*, and *Barnesiella*, with improved biological pathways in genetic information process and energy and calcium metabolism compared to PBS controls (Tang et al., 2025). Furthermore, CMT improves muscle characteristics (Cai et al., 2024), reduces prolonged light exposure-induced muscle injury (Yu et al., 2025) and increases immunity (Khalid et al., 2024) by changing the microbial profiles in recipients. The similar outcomes also revealed in chickens via FMT, increased growth performance (Cao et al., 2023; Ma et al., 2023; Liu et al., 2025; Xie et al., 2025; Xu et al., 2025), bone health (Bilal et al., 2025), and fermentative capability (Song et al., 2025; Zhao et al., 2026) as well as reduces bacterial infections (Wang et al., 2022; Pang et a., 2023). Taken together, the early postnatal (postpartum) period is a vital window for continuously developing of many biological systems including colonization of intestinal bacteria, and the early-life shaping the gut microbiota extremely influences the development of the gut microbial composition and function, consequently, affects brain function through the MGB axis, leading to a long-lasting impact on the responses in animals subjected to various stressful episodes. van der Eijk et al. (2020) further investigated if gut microbiota influences the development of feather pecking in chickens. In the study, mixed pooled luminal contents of the ileum, caeca, and colon collected from the high (HFP) or low (LFP) feather pecking adult birds (30-wk-old) with different microbial characteristics (a higher relative abundance of *Clostridiales* or *Lactobacilla*les in HFP and LFP, respectively) were transplanted into day-old chicks. It caused more active behavioral responses in both lines and lower 5-HT levels in LFP birds received HFP microbiota compared to relative controls (treated with saline). The findings reveal that early-life microbiota transplantation has long-term effects on behavioral and neuroendocrine characteristics, influencing development of feather pecking. It offers the potential for developing novel management practices to increase productivity and simultaneously to prevent physiological and behavioral disorders in chickens. However, it is still unclear how injurious behaviors are raised as a consequence of disrupted gut microbiota and its communication with the brain. Some studies have reported there were no differences in the cecal microbiota diversity and its major function in chickens when variations of confounding factors, such as breed and diet, were created (Cardenas et al., 2021). A recent study also reported that antagonistic behavior and feather pecking are not linked to gut microbiota composition and predicted function in laying hens (Borda-Molina et al., 2021). To fill the gaps, we used diversely selected inbred genetic lines to investigate if modulation of gut microbiota during early postnatal time can be an optimal method to prevent injurious behaviors in chickens (Dennis et al., 2004, 2014; Hu et al., 2022; Fu et al., 2022, 2023, 2024).

### Biological characteristics of the selected chicken lines and cecal microbiota transplantation in injurious behaviors

**Fig. 2:**
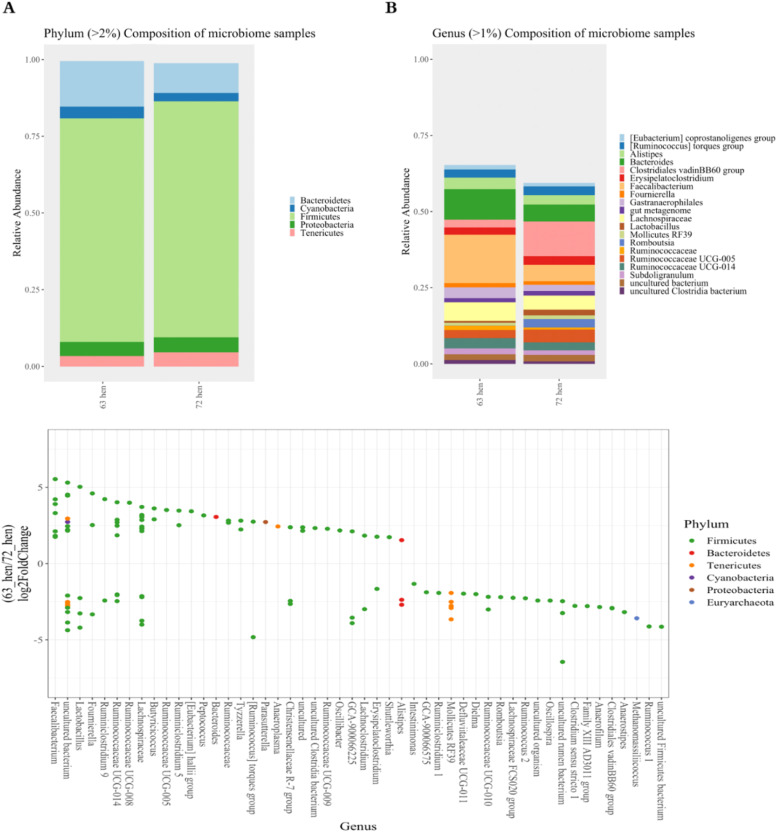
The different cecal bacterial compositions between two diversely selected chicken lines. (A) phyla, (B) genera analyses that compose an average of >2% and >1%, respectively, of the community, and (C) Pairwise comparisons (DESeq2 analysis) of differential abundant OTUs (p < 0.05) in lines 6_3_ and 7_2_. OTUs were assigned to genus (x-axis) and phylum level (colors). Positive “log2 Fold Change” values (y-axis) indicate higher abundance in 6_3-_hens, and negative values indicate higher abundance in 7_2_-hens. (Hu et al., 2022).

In a multi-year study, two divergently selected inbred chicken lines, 6_3_ and 7_2_, were used. The chicken lines have been continuously selected and maintained for resistance (line 6_3_) or susceptibility (line 7_2_) to Marek’s disease since the late 1960s (Bacon et al., 2000). As predicted, the selection has resulted in line differences in production with unique neuroendocrine function, immunity, and behavioral appearance (Heidari et al., 2016; Hu et al., 2022). Compared to 6_3_ chickens, 7_2_ chickens have larger sizes of primary lymphoid organs (i.e., the bursa of Fabricius and the lobes of the thymus) (Zhang et al., 2006) with suppressed cellular immunity, as a higher number of CD4 T cells (helper T lymphocytes coordinating the immune response against infection) but a lower number of CD8 T cells (cytotoxic T lymphocytes directly defending against intracellular pathogens and cancer cells) (Yu et al., 2011: Heidari et al., 2016). In responding to Marek’s disease virus challenge, 7_2_ chickens have higher levels of IL-6 and IL-18 mRNA expressions (pro-inflammatory cytokines), while 6_3_ chickens have higher gene expressions of IL-8 (known as monocyte-derived neutrophil chemotactic factor and angiogenic cytokine) as well as toll-like-receptors (TLR)-3 and TLR-7 (mediators of inflammatory pathways between the adaptive immunity and the innate immunity) (Kaiser et al., 2003; Haunshi and Cheng, 2014). Furthermore, 7_2_ chickens show high stress levels in social contexts evidenced by a higher heterophil to lymphocyte (H/L) ratio and plasma corticosterone level with more aggressive pecks and longer durations of fights correlated with lower levels of brain 5-HT (Dennis et al., 2004; Dennis and Cheng, 2014; Hu et al., 2022) (Table 1A,B). The selection also causes differences in cecal bacterial diversity. Line 6_3_ chickens have enriched levels of several genes including *Bacteriodes, Butyricicoccus, Faecalibacterium, Oscillibacter*, and *Ruminococcaceae UCG*-005, -008, -009 in the ceca, while line 7_2_ chickens dominate with genes including *Clostridiales vadin* BB60 (Fig. 2). The outcomes of predicted bacterial functional gene analysis reveal that the KYN pathway of TRP metabolism was upregulated in line 7_2_ chickens, indicated by the brain 5-HT levels were negatively correlated with the *Clostridiales vadin* BB60 group; while TRP-5HT activity was inherently higher in line 6_3_ chickens (Hu et al., 2022). Taken together, social sensitivity and injurious behaviors of the selected chickens are regulated through both the HPA axis and serotonergic system, which are similar to those in humans and other mammals.

**Table 1:** A. Central serotonergic metabolism in the raphe nucleus; and B. Peripheral serotonergic metabolism and stress indicators, corticosterone and heterophil/lymphocyte ratio, between the divergently selected 6_3_ and 7_2_ inbred chicken lines.

A.				
Treatment	TRP (ng/g)	5-HT (ng/g)	5-HIAA (ng/g)	5-HIAA/5-HT
Line 6_3_	1183.8^a^	512.6^a^	151.8	0.30^b^
Line 7_2_	963.2^b^	382.7^b^	168.9	0.44^a^
SEM	22.4	8.2	12.9	0.02
p-value	0.08	0.01	0.62	0.04

B.				
Treatment	TRP (ng/g)	5-HT (ng/g)	CORT (ng/mL)	H/L ratio
Line 6_3_	171.5^a^	61.4	8.4^b^	0.16^b^
Line 7_2_	121.4^b^	59.5	9.8^a^	0.50^a^
SEM	15.4	3.8	0.5	0.04
p-value	0.03	0.73	0.05	<0.0001

^a,b^ Least squares mean within a column for the 2 lines lacking a common superscript differ (p < 0.05). Lines 6_3_ and 7_2,_ inbred lines selected for resistance or susceptibility to Marek’s disease. 5-HT, serotonin; 5-HIAA, 5-hydroxuindoleacetic acid; 5-HT/5-HIAA, CORT, corticosterone; H/L ratio, heterophil-to-lymphocyte ratio; serotonin metabolic index; TRY, tryptophan. (Hu et al., 2022)

**Fig. 3:**
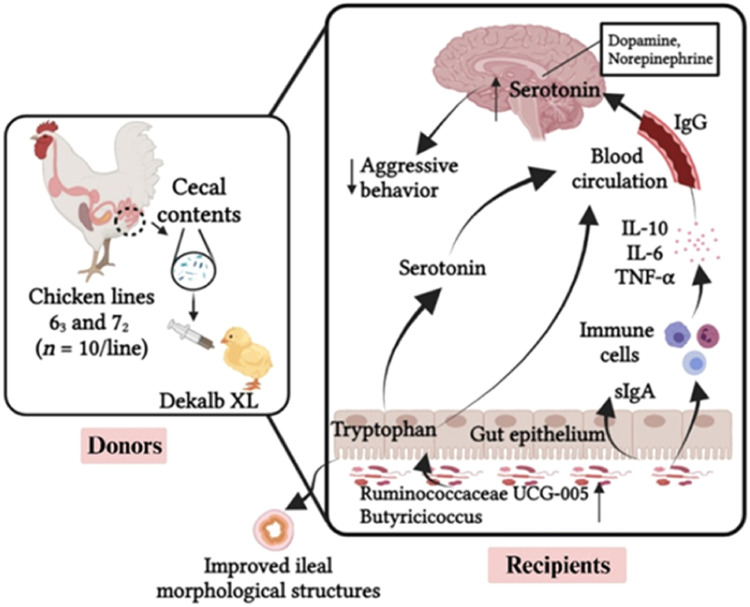
The experimental design and the proposed mechanisms underlying the effects of cecal microbiota collected from the diversely selected donor lines on health and behavior of recipient chickens (Prepared with BioRender.com. Fu et al., 2022,).

To examine if injurious behaviors in chickens can be inhibited via modifying gut microbiota, early-life CMT was conducted on the selected lines (6_3_ and 7_2_) since 2021 (Hu et al., 2022; Fu et al., 2022, 2023, 2024) (Fig. 3). In the study, the cecal contents were separately collected from adult hens of the selected lines (6_3_ and 7_2_ donors, at 60-wk-old), then orally transferred to day-old male chicks of Dekalb XL white commercial line (recipients), with 3 treatments for a 16-week trial. Male chicks were used in the study as that there is the gender-dependent difference in aggression in humas and various animals including chickens, i.e., males generally are more involved in aggressions than females, especially physical aggression (Fahlgren et al., 2022; McCurry et al., 2025) due to the differences in the hormones (such as the levels of testosterone) (George and Rosvall, 2022; Lin et al., 2024) and physical-anatomical specialization (such as the mass and strength of the musculoskeletal system) (Petrie et al., 2023; Plotkin et al., 2024). The most important outcome of the study is that compared to 6_3_-CMT recipients, 7_2_-CMT recipients showed higher aggression with a lower serotonergic activity (lower concentrations of hypothalamic 5-HT and 5-HIAA), which is similar to the physiological and behavioral characteristics of the donors of line 7_2_ (Fu et al., 2022, 2024). Specifically, 7_2_-CMT chickens had a higher phylogenetic diversity in the ceca, which could be linked to the different susceptibility to Marek’s disease between the 6_3_ and 7_2_ donors. Compared to 7_2_-CMT recipients, 6_3_-CMT recipients have an abundant ASV (Amplicon Sequence Variant) belonging to *Ruminococcaceae UCG*-005, a genus that has been reported functionally as SCFAs producers (Fu et al., 2023). Gut microbiota-derived SCFAs have essential effects on each component of the MGB axis reducing social stress in chickens via manipulating hypothalamic function and 5-HT signaling by acting on 5-HT transporter (SERT) and receptors (Dalile et al., 2020; Liu et al., 2021; Jadhav et al., 2022; de Sousa et al., 2025). The results could indicate that CMT directly and/or indirectly influences the syntheses of neuropeptides, and neurotransmitters, including TRP and 5-HT, and the exhibition of aggressive behavior (Cheng et al., 2024; Mavros et al., 2024). Taken together, the current results indicate there is a donor-recipient interaction at chicken physiological and behavioral stages of development. CMT conducted at an early age reduces aggressive behavior in recipients according to the donors’ behavioral and biological phenotype via regulating the development of the cecal microbiota composition, consequently affecting the hypothalamic serotonergic activity and stress reaction. These findings demonstrate that early-life modification of gut bacterial community have long-term effects on chicken health and welfare by reducing injurious behaviors.

## Conclusion

Gut microbiota functions as a neuroendocrine organ releasing various immunomodulators and bioactive factors with critical roles in early neuronal programming and later response to various stimulations in animals including chickens. The current results show that early-life CMT from the donor hens of the two divergently selected inbred lines reduces injurious behaviors in male days-old recipients through regulating the development and function of the MGB axis via alterations of cecal microbiota composition, hypothalamic serotonergic activity, and stress reaction. The outcomes could provide new insights into uncovering mechanisms underlying the gut microbiota in regulating stress-induced abnormal behavior and providing strategies for developing biotherapeutic interventions to reduce injurious behaviors in chickens. To fully develop and test the potential strategy, a comprehensive study is required to further explore the multifactorial nature of cecal microbiota transplantation in injurious behaviors and its scope of application, especially in laying hens.

## Funding

This study was supported by the grant award (No: 2017-67015-26567) of the NIFA-AFRI, USDA.

## CRediT authorship contribution statement

**Heng-wei Cheng:** Writing – review & editing, Writing – original draft, Formal analysis, Conceptualization.

## Disclosures

The author declares no conflicts of interest.
